# Research on the Hot Straightening Process of Medium-Thick Plates Based on Elastic–Viscoplastic Material Modeling

**DOI:** 10.3390/ma17102385

**Published:** 2024-05-16

**Authors:** Zhanyuan Xue, Ben Guan, Yong Zang

**Affiliations:** 1 School of Mechanical Engineering, University of Science and Technology Beijing, Beijing 100083, China; 2 Shunde Innovation School, University of Science and Technology Beijing, Foshan 528399, China

**Keywords:** hot straightening, medium-thick plate, temperature, strain rate, numerical calculation, viscoplastic constitutive model, cross-sectional continuous reverse bending model, mechanical parameter behavior

## Abstract

With the continuous improvement in the strength of medium-thick plate materials, the hot straightening of plates at high temperatures is increasingly influencing the final defect characteristics of products. In the high-temperature hot straightening process, the temperature and straightening speed of the plate significantly influence its intrinsic material properties, which, in turn, affect the straightening characteristics of the plate. However, most current material models used in the straightening process do not consider the relationship between temperature and strain rate, which leads to an inaccurate characterization of the actual material structure. Additionally, the continuous reverse bending mechanics model for straightening does not account for the impact of different bending strain rates on the bending characteristics of the plate in the thickness direction. In this study, a numerical calculation method was employed to investigate the evolution process of stress and curvature in the roll-type hot straightening process of medium-thick plates. Experimental data and mathematical methods were utilized to develop a viscous plastic material model that accounted for temperature and strain rate. Furthermore, a cross-sectional continuous reverse bending model was established, taking into account the temperature and straightening speed, enabling a reasonable interpretation of the mechanical parameter behaviors of medium-thick plates during high-temperature straightening.

## 1. Introduction

The hot straightening process is a crucial step in finishing medium-thick plates, involving high-temperature hot deformation conditions ranging from 450 °C to 900 °C [1]. It is the final hot deformation process in the production of plates and, together with the subsequent cold straightening process, it plays a vital role in regulating the final geometric shape and internal stress state of the plates [2,3,4]. Traditionally, the hot straightening process was considered an intermediate rough finishing process, with further evolution of the plate shape during the cooling process. The final shape of the plate was adjusted through cold straightening and other room-temperature finishing processes. However, with the improvements in plate strength and the widespread adoption of controlled rolling and cooling technology, the necessary straightening force and the accumulation of defects in the production process have significantly increased. This presents challenges in effectively eliminating any remaining defects after hot straightening during subsequent room-temperature finishing processes. As a result, the precision and control of the hot straightening process need to be enhanced, necessitating a more accurate analysis and prediction model for the process [5]. Consequently, the demand for accurate regulation in the hot straightening process of medium-thick plates is continuously rising.

After analyzing the deformation characteristics of the hot straightening process of medium-thick plates, it becomes evident that this process exhibits unique plastic deformation characteristics, including high-temperature hot deformation, small deformation volume, and continuous reverse bending loading. The high-temperature hot deformation characteristics differentiate it from the cold straightening process carried out at room temperature. This leads to pronounced temperature and strain rate-dependent viscoplastic deformation characteristics in the medium-thick plate material. Moreover, the small deformation and continuous reverse bending loading characteristics distinguish it from the typical large deformation and unidirectional loading observed in plastic hot processing. Consequently, there are significant differences in the macroscopic viscoplastic deformation behavior and characterization of the plates. Industrial experience over an extended period has further revealed that the hot straightening temperature, straightening speed, and other process parameters significantly impact the effectiveness of the hot straightening process. Therefore, accurately describing and characterizing the viscoplastic deformation behavior of medium-thick plates in the hot straightening process is a crucial problem to address in order to gain a comprehensive understanding of the process and establish a precise analytical model for the hot straightening process of medium-thick plates.

A significant number of researchers, both domestic and international, have conducted extensive systematic analyses and research on the process of straightening [6,7,8,9,10,11,12,13,14,15,16,17,18,19,20,21,22,23,24,25,26,27,28,29,30]. In order to analyze the straightening process, finite element numerical simulation and the curvature integral method have been widely employed as numerical simulation techniques. Gribkov et al. [6] developed a finite element model using simulation to investigate roll straightening. They determined that the contact relationship between the plate and the straightening roll and also the yield strength of the plate material are the boundary conditions that affect the maximum straightening roll pitch in the roll straightening machine. Jian-liang Sun et al. [10] proposed a curvature integral method taking into account the variation in the thickness of the cross-section of longitudinal profiled plates and the dynamic reduction of straightening rolls. The authors utilized an analytical model that combined the integral method with a linearly decreasing straightening scheme to investigate the straightening process of longitudinal profiled plates. Kotov et al. [13] developed a finite element model of hot strip roll straightening to investigate the stress state and residual stress distribution during the roll straightening process of hot strips. However, numerical analysis has often failed to adequately explain the continuous reverse bending deformation behavior of plates during the straightening process. In our research on the continuous reverse bending model of the straightening process, our research team [14] proposed a continuous reverse bending solution model that considers the geometric behavior of cross-section stress. Liu Dongye [26] further analyzed the reverse bending characteristics of plastic-reinforced materials during the straightening process. Later, Gui Hailian [27] and Ma Xiaobin [28] provided detailed demonstrations of the influence of the Bauschinger effect on the deformation behavior of the plate straightening process using various material modeling approaches. Notably, Zhou Jialin and other scholars [29,30] highlighted the viscoplasticity characteristics in the heat straightening process and established a corresponding viscoplasticity-only isomorphic model of medium-thick plate material through experiments, providing valuable insights for the development of this study. However, there is still room for improvement in accurately describing the macroscopic heat straightening process of the plate. It is evident that previous scholars have made significant efforts to enhance the analysis of the mechanical behavior of the plate-straightening process by refining the description of the material’s structural relationships. However, most of the existing research focuses on the elastic–plastic deformation of the material in the cold straightening process. The analysis of the material’s viscoplastic behavior still depends on the elastic–plastic reverse bending mechanical model. This approach falls short of accurately describing the mechanical behavior of the hot straightening process for medium-thick plates. Thus, this paper aims to investigate the stress and curvature evolution in the hot straightening process of medium-thick plates and establish a cross-section continuous bending model that comprehensively considers temperature and straightening speed. The objective is to develop an analytical model that accurately represents the mechanical behavior of the hot straightening process for medium-thick plates.

## 2. Numerical Solution Modeling

### 2.1. Modeling the Continuous Reverse Bending Solution for a Medium-Thick Plate Section

**Figure 1:**
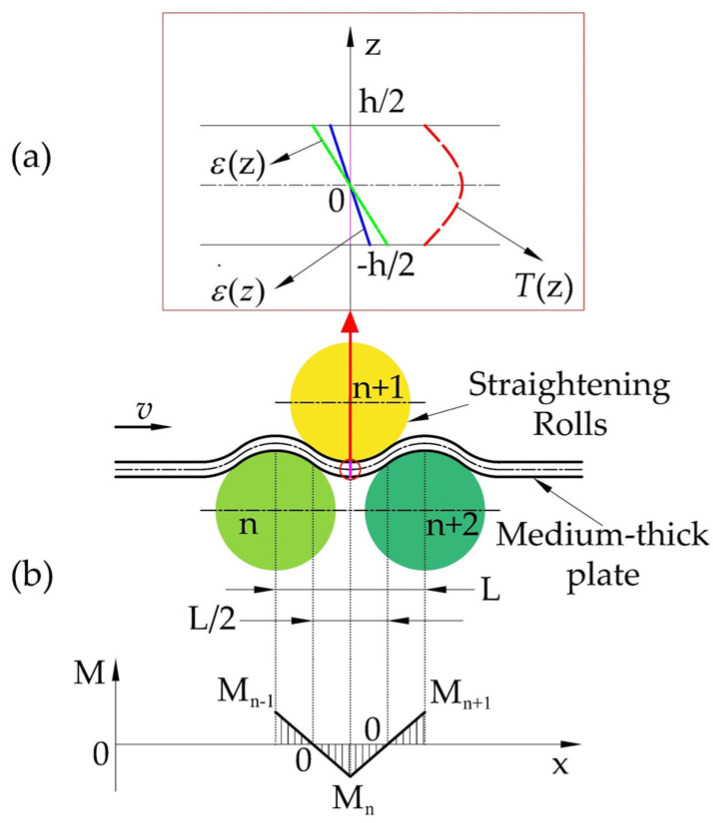
A schematic diagram illustrating the distribution of temperature and strain rate in the hot-straightened cross-section of a medium-thick plate.

The temperatures and strain rates at each position along the bending section of the plate in the hot straightening process significantly influence its stress state. Therefore, the cross-section continuous reverse bending solution model for the hot straightening process for plates must consider the distribution of temperature and strain rate along the bending section. As depicted in Figure 1a, the strain in the thickness direction of the plate increases linearly from 0 at the neutral layer to the outer layer, leading to a linear increase in strain rate at each position of the bending cross-section. The surface of the plate undergoes controlled or natural cooling before hot straightening, resulting in a gradual decrease in temperature within the bending cross-section from the center to the outer regions.

#### 2.1.1. Mathematical Description of Bending Strain Rate Distribution in a Cross-Section

As depicted in Figure 1b, the distribution of bending strain rate in the cross-section was quantitatively characterized by setting the plate straightening speed to ***v***, the distance between the straightening rollers to *L*, and identifying the maximum bending strain and bending moment at the point where the plate is in contact with the vertical underneath the straightening rollers. Based on previous studies [31], it can be approximated that the midpoint between the upper and lower straightening rollers represents the point of zero bending moment, which corresponds to a bending strain of 0 at each position along the cross-section. Thus, the time required for the cross-section at each position to transition from a bending strain of 0 to the maximum strain is denoted as $t=\frac{L}{4v}$. Consequently, the strain rate distribution across the cross-section was determined by the time taken for each location to reach the maximum strain from a bending strain of 0.

(1) $$\varepsilon \left(z\right)=Cz\begin{array}{cc} & \left(-h/2\leq z\leq h/2\right) \end{array}$$

(2) $$\dot{\varepsilon }\left(z\right)=\frac{4\varepsilon \left(z\right)v}{L}$$

In Equations (1) and (2), *ε* represents the bending strain of the cross-section at a specific position along the thickness direction, *C* represents the bending curvature, while *z* indicates the coordinate of the cross-section along the thickness direction. The significance and range of *z* remain the same in the following formulas. Additionally, “*h*” refers to the thickness of the plate.

#### 2.1.2. Mathematical Description of Cross-Sectional Temperature Gradients

In the hot straightening process, the change in cross-section temperature is negligible because of the short duration and the absence of strong convective heat transfer conditions. Therefore, the temperature distribution of the plate can be simplified by considering it as an adiabatic process. This indicates that the plate’s temperature is solely determined by the temperature field formed during its pre-cooling process.

According to a previous study [32] that analyzed finite element simulation data of the heat transfer model of the cross-section during the cooling process of hot rolled sheets, the temperature distribution along the cross-section follows an approximate parabolic shape starting from the bending neutral layer and extending outward. This temperature distribution is represented by the red line in Figure 1a. As a result, the temperature distribution of the bending section can be effectively described using a quadratic function, as follows:

(3) $$T(z)=T_{0}-kz^{2}$$

In Equation (3), the temperature coefficient (*k*) is designated and is directly linked to the pre-cooling conditions of the plate, and *T*_0_ represents the temperature of the bending neutral layer within the cross-section.

#### 2.1.3. Modeling of Cross-Section Continuous Reverse Bending

The viscoplastic material model of the cross-section of the continuous reverse bending, as shown in Figure 1b, was considered for the nth roll to the n + 2 rolls between the nth bending. To illustrate this, let *C*_n_ represent the curvature of the nth reverse bending, ${\sigma_{c}}^{\left(n-1\right)}(z)$ be the cross-section of the residual stress after the n − 1th bending, which causes the residual strain ${\varepsilon_{c}}^{\left(n-1\right)}(z)=\frac{{\sigma_{c}}^{\left(n-1\right)}(z)}{E}$, and ${\sigma_{w}}^{n}(z)$ be the bending stress produced during the nth bending process. Through considering the geometric coordination of the bending process deformation and the superposition of the residual strain, the fundamental equation for the theoretical bending stress–strain in the elastic deformation region, without considering the yield condition of the stress, can be written as:

(4) $$\varepsilon^{\left(n\right)}(z)={\varepsilon_{w}}^{\left(n\right)}(z)+{\varepsilon_{c}}^{\left(n-1\right)}(z)$$

(5) $${\sigma_{e}}^{\left(n\right)}(z)=E\varepsilon^{\left(n\right)}(z)$$

In Equations (4) and (5), the bending strain formed by the nth bending alone is represented as ${\sigma_{w}}^{n}(z)$. ${\sigma_{e}}^{\left(n\right)}(z)$ denotes the loading stress in the elastic deformation zone, while E refers to the elastic modulus of the material.

The loading stress ${\sigma_{ep}}^{\left(n\right)}(z)$ in the plastic zone under the viscoplastic state is determined by the degree of deformation *ε*^(*n*)^(*z*), the strain rate ${\overset{\cdot }{\varepsilon }}^{\left(n\right)}(z)$, and the deformation temperature *T(z*), as demonstrated in Equation (6). The detailed explanation and solution procedure can be found in Section 2.2.

(6) $${\sigma_{ep}}^{\left(n\right)}(z)=f(\varepsilon^{\left(n\right)}(z),{\overset{\cdot }{\varepsilon }}^{\left(n\right)}(z),T(z))$$

The three terms on the right-hand side of Equation (6) correspond to Equations (1)–(3) for the nth bending, respectively.

The evaluation of *ε*^(*n*)^(*z*) is affected by the residual stress due to the previous bending rebound, which prevents it from returning to its initial position. Therefore, the judgment of the elastic limit strain *ε*_t_ needs to consider this impact. If $\left|\varepsilon^{\left(n\right)}(z)\right|>\varepsilon_{t}$, the value of $\sigma^{\left(n\right)}(z)$ is assigned as $\sigma^{\left(n\right)}(z)=\pm {\sigma_{ep}}^{\left(n\right)}(z)$; if $\left|\varepsilon^{\left(n\right)}(z)\right|<\varepsilon_{t}$, the value of $\sigma^{\left(n\right)}(z)$ is assigned as $\sigma^{\left(n\right)}(z)={\sigma_{e}}^{\left(n\right)}(z)$, to obtain the stress distribution of the cross-section loaded in bending at the nth position. The residual stress resulting from $\varepsilon^{\left(n\right)}(z)$ previous bending rebound prevents it from returning to its initial position. The residual stress affects the strain assessment, thus impacting the evaluation of the elastic limit strain *ε_t_*. It is important to consider this impact, assigning the value of $\sigma^{\left(n\right)}(z)=\pm {\sigma_{ep}}^{\left(n\right)}(z)$ to $\left|\varepsilon^{\left(n\right)}(z)\right|>\varepsilon_{t}$ and the value of $\sigma^{\left(n\right)}(z)={\sigma_{e}}^{\left(n\right)}(z)$ to $\left|\varepsilon^{\left(n\right)}(z)\right|<\varepsilon_{t}$. Consequently, the stress distribution under the nth bending of the cross-section can be obtained.

After deriving the equations for the stress distribution in the cross-section during complex viscoplastic bending, the bending moments can then be calculated as follows:

(7) $$M^{\left(n\right)}=\frac{3}{2\sigma_{s}}\int_{-1}^{1}\sigma^{n}(z)\cdot z\cdot dz$$

In Equation (7), $M^{\left(n\right)}$ represents the nth bending moment of the section and *σ_s_* denotes the ultimate yield stress of the section.

The equation for stress distribution in the rebound is as follows:

(8) $${\sigma^{\left(n\right)}}^{\prime }(z)=\frac{-M^{\left(n\right)}C_{n}z}{I\varepsilon_{s}}$$

In Equation (8), ${\sigma^{\left(n\right)}}^{\prime }(z)$ represents the rebound stress of the nth bending of the section, *I* denotes the section’s moment of inertia, and *ε_s_* signifies the ultimate yield strain of the section.

The equation for the distribution of residual stress is as follows:

(9) $${\sigma_{c}}^{\left(n\right)}(z)={\sigma_{w}}^{\left(n\right)}(z)+{\sigma^{\left(n\right)}}^{\prime }(z)$$

The equation for the residual curvature of the section after rebound is as follows:

(10) $$C_{C_{n}}=C_{n}-\frac{M^{\left(n\right)}C_{n}}{EI\varepsilon_{s}}$$

In Equation (10), $C_{C_{n}}$ represents the residual curvature of the section after the nth bending spring-back.

**Figure 2:**
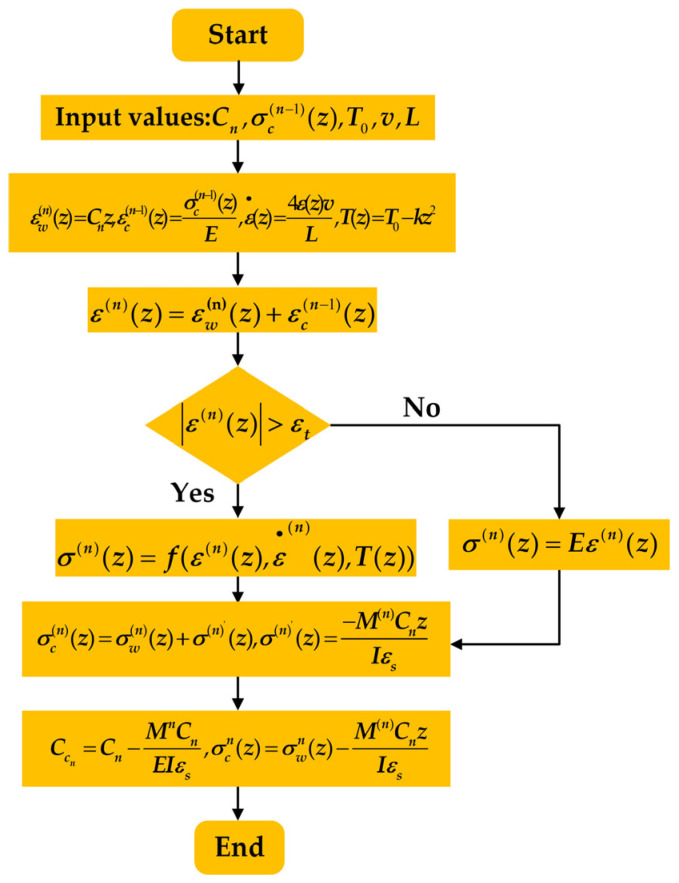
Logic block diagram for numerical analysis model of bending of viscoplastic materials.

This study applied the principle of residual strain–stress superposition, the static equilibrium relationship, and the criteria for determining the yield condition of the cross-section to derive Equations (1)–(10) for the numerical analysis of the nth viscoplastic bending process of the cross-section. The specific steps for the numerical analysis are referenced from the literature [25]. The logical block diagram of the numerical analytical model for the viscoplastic material is illustrated in Figure 2.

### 2.2. Establishment of the Principal Structural Model for Medium-Thick Plate Materials

#### 2.2.1. Mathematical Description of Viscoplastic Behavior of Materials

According to the literature [33], the conventional flow stress model accurately describes the initial stress-hardening and dynamically restored deformation behavior of metals before reaching the peak stress. It considers the effects of deformation temperature (*T*), strain rate ($\overset{\cdot }{\varepsilon }$), and degree of deformation (*ε*) on the flow stress (*σ*). The deformation of hot straightening under single unidirectional loading is characterized by strain hardening as the dominant viscoplastic hot deformation, which includes dynamic recovery. Since the maximum plastic strain in this process is only 5%, the viscoplastic deformation behavior of medium-thick plate material under hot straightening unidirectional loading state can be expressed as follows:

(11) $$\sigma =A\varepsilon^{m}{\overset{\cdot }{\varepsilon }}^{i}\exp(QT+D\varepsilon )$$

In Equation (11), *σ* represents the flow stress. The right-hand side of the equation consists of three elements. The first element, *ε^m^*exp (*Dε*), quantifies the degree of deformation on the flow stress using a power function. Here, *m* corresponds to the strain hardening index, while D is the regression coefficient that depends on the grade of steel. The second element, ${\overset{\cdot }{\varepsilon }}^{i}$, indicates a logarithmic relationship between the strain rate and the flow stress, with *i* representing the strain rate hardening index. Furthermore, the temperature of the deformation is also a factor in this relationship. Finally, the third term, *A*exp(*QT*), demonstrates an exponential relationship between the flow stress and the temperature of deformation. The regression coefficients *A* and *Q* are influenced by the grade of steel.

#### 2.2.2. Description of the Elastic Behavior of Materials

The straightening process of medium-thickness plates involves a continuous bending and rebounding process. It is crucial to accurately characterize the loading and unloading behavior of the material. During the unloading process, the elastic behavior of the viscoplastic material becomes predominant. Thus, it is necessary to explicitly describe the material’s modulus of elasticity and the elasticity–viscoplasticity demarcation point under different conditions.

The elastic modulus of metallic materials primarily depends on interatomic forces and shows temperature sensitivity. In a study investigating the material properties of high-temperature Q345 steel and its influencing factors [34], a mathematical model was developed to describe the relationship between the elastic modulus *E* and temperature *T*:

(12) $$E=a+bT+cT^{2}+dT^{3}$$

In Equation (12), the coefficients *a*, *b*, *c*, and *d* represent the elastic modulus to be determined.

During the hot straightening process, the material quickly enters the viscoplastic deformation stage with minimal strain. It is challenging to determine the exact elastic–viscoplastic transition point of the material based solely on experimental data. Therefore, we defined the stress value corresponding to 0.2% strain in the principal structure as the initial stress of the material’s viscoplasticity (*σ*_0.2_). This stress value served as the elastic–viscoplastic transition point, also known as the yield limit stress. Consequently, the corresponding interfacial strain is as follows:

(13) $$\varepsilon_{t}=\frac{\sigma_{0.2}}{E}$$

#### 2.2.3. Viscoplastic Material Model of Q345 Medium-Thick Plate

Q345 steel obtained from a plate mill was selected as the study material, and its chemical composition is outlined in Table 1. The stress–strain relationship associated with the viscoplastic material was derived by integrating experimental data with the calculated values in the model.

In this paper, a single-pass low-temperature hot simulation tensile experiment was conducted on a Q345 steel plate using a Gleeble-1500 hot simulator. The hot straightening temperature for medium-thickness plates typically ranges from 400 °C to 800 °C, the strain rate falls within the range of 0.005 s^−1^ to 0.5 s^−1^, and the experimental program aimed to simulate hot straightening deformation conditions. The specimen plate was heated at a rate of 10 °C/s until it reached 1000 °C, where it was held for 10 min. Subsequently, it was cooled at a rate of 5 °C/s to temperatures ranging from 450 °C to 650 °C, where it underwent tensile deformation at strain rates of $\dot{\varepsilon }=0.01{\;s}^{-1}$ and $\dot{\varepsilon }=0.1{\;s}^{-1}$. Stress–strain data were recorded at various temperatures and strain rates. Table 2 illustrates the specific experimental parameters for this program.

Parameterization of the viscoplastic material model corresponding to Equations (11)–(13) based on the results of the hot tensile experiments was calculated as follows.

Applying logarithms on both sides of Equation (11) yields:

(14) $$\mathrm{In}\sigma =\mathrm{In}A+m\mathrm{In}\varepsilon +i\mathrm{In}\dot{\varepsilon }+QT+D\varepsilon$$

**Figure 3:**
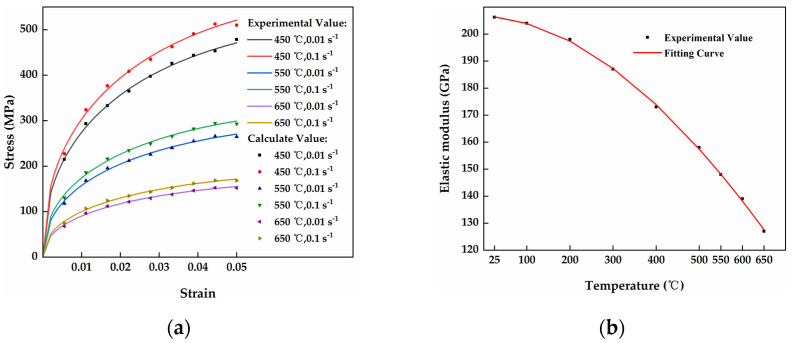
Comparison of experimental and calculated values in the model of the viscoplastic material in a Q345 medium-thick plate. (**a**) Comparison of experimental and calculated values of flow stress in a Q345 medium-thick plate; (**b**) Measured values of modulus of elasticity in a Q345 medium-thick plate at different temperature intervals.

The experimental values for temperatures of *T* = 450 °C, $\overset{\cdot }{\varepsilon }=0.01/0.1{\;s}^{-1}$ and *T* = 650 °C, $\overset{\cdot }{\varepsilon }=0.01/0.1{\;s}^{-1}$ from Figure 3a were used in Equation (14) for multivariate linear fitting. The fitting results produced the following parameter values: *A* = 11,382.1, *m* = 0.4352, *i* = 0.0438, *Q* = −0.0037, *D* = −3.9797. Thus, the specific expression for model of the viscoplastic material in the Q345 high-strength plate is as follows:

(15) $$\sigma =11382.1\varepsilon^{0.4352}{\dot{\varepsilon }}^{0.0438}\exp\left[-(0.0037T+3.9797\varepsilon )\right]$$

According to Equation (12), the measured values of the elastic modulus at various temperature intervals underwent nonlinear regression calculations, as illustrated in Figure 3b. The regression coefficients for the model were determined to be *a* = 206.3, *b* = −0.02063, *c* = 1.444 × 10^−4^, and *d* = −6.18 × 10^−7^. In addition, the regression exponent (R^2^) had a value of 0.99932, approximately equal to 1. Based on these calculations, the specific expression for the obtained elastic modulus model is as follows:

(16) $$E=206.3-0.02063T+1.444\times 10^{-4}T^{2}-6.18\times 10^{-7}T^{3}$$

Based on the formulation of Equation (13), the elastic–plastic strain cutoff point *ε*_t_ was determined for different elastic moduli corresponding to various temperatures. Consequently, the stresses in the elastic phase of the viscoplastic material model at various temperatures and strain rates were obtained, as depicted in Table 3.

The data taken at *T* = 550 °C, $\overset{\cdot }{\varepsilon }=0.01{\;s}^{-1}$ and *T* = 550 °C, $\overset{\cdot }{\varepsilon }=0.1{\;s}^{-1}$, which were not included in the regression analysis of the aforementioned model, were used to validate the accuracy of the model. The data points were substituted into the intrinsic equation provided in Equation (15) to determine the flow stress value of the Q345 high-strength plate under these conditions. The comparison between the experimental values and the model’s calculated values is depicted in Figure 3a. As shown in Figure 3a, the experimental values closely aligned with the calculated values, indicating a high level of accuracy in the model’s calculations.

Based on the fitted viscoplastic material model, the elastic and plastic segments were combined to form the complete viscoplastic material model for calculating the flow stress at different temperatures (*T*) and strain rates ($\dot{\varepsilon }$). The results are presented in Figure 3a. As shown in the figure, a higher temperature (*T*) resulted in a lower flow stress value when the strain rate ($\dot{\varepsilon }$) was kept constant. Additionally, the difference in stress values was larger at higher temperatures. Conversely, when the temperature (*T*) remained constant, increasing the strain rate ($\dot{\varepsilon }$) resulted in higher flow stress values, albeit with smaller variations in stress levels. This indicates that both temperature (*T*) and strain rate ($\dot{\varepsilon }$) influence the stress calculation of the material model, with temperature having a greater impact on flow stress compared with strain rate. Consequently, considering the experimental and calculated values discussed above, the material model is deemed suitable for the hot straightening process, as it effectively incorporates the effects of temperature and strain rate.

### 2.3. Mechanical Characterization of Different Material Models through Cross-Section Bending

After developing a numerical analytical model for the continuous buckling of a cross-section under the viscoplastic material model, considering temperature and strain rate, it was imperative to establish a comparable model of continuous buckling without considering these factors.

#### 2.3.1. Modeling of Cross-Section Continuous Backbending Based on Linear–Elastic Ideal Plastic Materials

**Figure 4:**
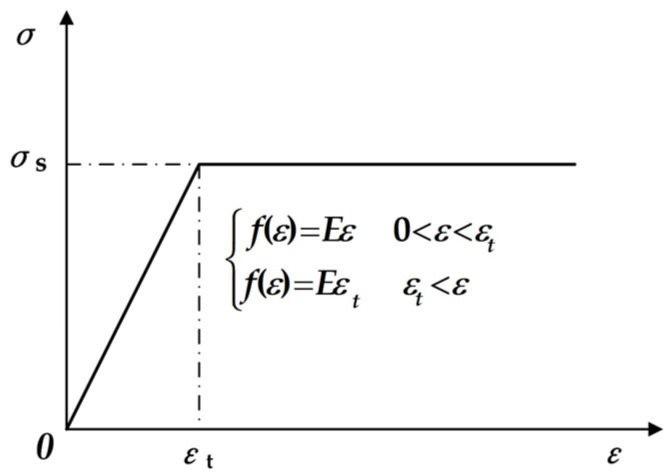
Schematic diagram of linear–elastic ideal plastic stress–strain relationship curve.

The study focuses on the linear–elastic ideal plastic material model [35]. Its stress–strain relationship is depicted in Figure 4, with the expression *σ* = *f* (*ε*, *ε*_t_, *σ*_s_, *E*). Following the logical framework of the numerical analysis presented in Section 2.1.3 for bending of the viscoplastic material model, the theoretical bending strain was determined by satisfying Equations (4) and (5) within the elastic deformation range, neglecting any influence of temperature and strain rate distribution on the cross-section.

Plastic zone in linear–elastic ideal plastic state: $\sigma^{\left(n\right)}(z)=\sigma_{s}$.

**Figure 5:**
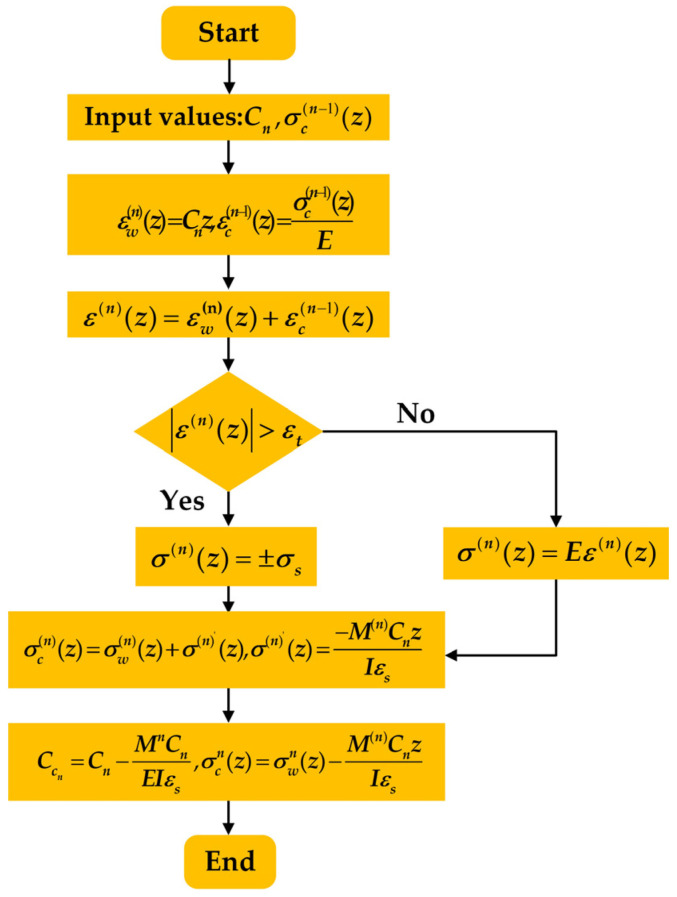
Logic diagram for the numerical analysis of a linear-elastic ideal plastic material model during bending.

After deriving the cross-sectional stress distribution equation for linear–elastic ideal plastic bending, the bending moment was calculated, and it complied with Equation (7). Similarly, the equation for the rebound stress distribution satisfied Equation (8), and the residual stress distribution equation aligned with Equation (9). Through analyzing the loading and rebound stress distribution following the nth bending of the linear–elastic ideal plastic material model, the numerical investigation of the continuous reverse bending of the cross-section in the material model was completed. Figure 5 illustrates the logical block diagram of the linear–elastic ideal plastic numerical mechanical analysis model.

#### 2.3.2. Stress Distribution during Cross-Section Bending with Different Material Models

Based on a previously established numerical analysis model of cross-section bending for various material models, this study examined and explored the mechanical behavior and bending characteristics of cross-section backbending. The research material chosen was Q345 steel plate with a plate section thickness of H = 0.02 m and a width of B = 1 m. The mechanical properties of the cross-section were numerically analyzed under the straightening conditions of temperature *T*_0_ = 500 °C, straightening speed *v* = 0.8 m/s, and roller distance *L* = 0.36 m. The analysis of the mechanical properties of the cross-section was conducted using a numerical analysis model for cross-section bending, based on various established material models.

**Figure 6:**
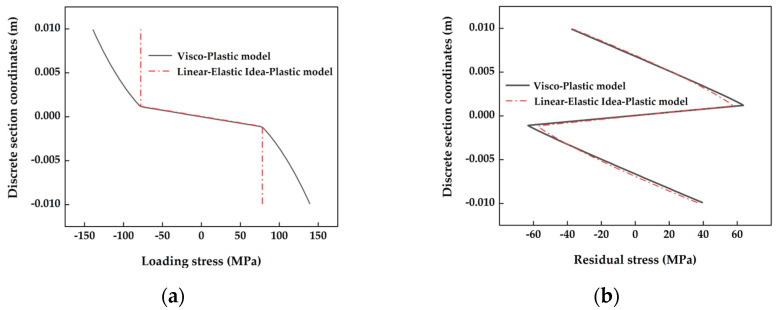
Typical cross-sectional stress distribution under various material models. (**a**) Schematic of loaded stress distribution in cross-section for various material models; (**b**) schematic distribution of cross-sectional residual stresses for various material models.

The initial curvature, *C*₀ = 0.2 m⁻¹, was selected as the standard bending cross-section for straightening, and the loading curvature for backbending, *C*_w_ = 0.5 m⁻¹,was analyzed numerically. This analysis was based on linear–elastic ideal plastic and viscoplastic materials. The stress distribution in the cross-section during the bending process is illustrated in Figure 6a. It was observed that the loading stresses corresponding to both the linear–elastic ideal plastic and viscoplastic models were similar in the elastic section. In the plastic section, the loading stresses for the linear–elastic ideal plastic material model remained relatively constant. In contrast, for the viscoplastic material model, the loading stresses increased non-linearly along the section’s edge direction. This discrepancy was primarily attributed to the differing stress descriptions between the two material models in the plastic stage.

The residual stress distribution following rebound is depicted in Figure 6b, showing that both models exhibited uniformly distributed residual stresses with minimal difference. This indicates that the rebound amount upon the material entering the plastic section was influenced by the interaction of material properties and various parameters within the model.

#### 2.3.3. Moment–Curvature Curves of Cross-Section Bending Processes under Different Material Models

**Figure 7:**
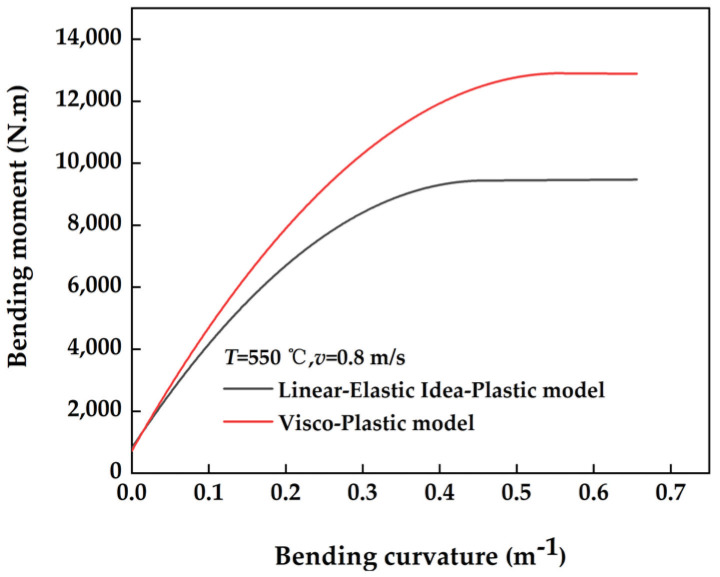
Moment-curvature curves for different material models.

The bending characteristics of a section during elastic–plastic bending are primarily represented by the moment–curvature relationship during loading. Four distinct reverse bending loading curvatures, namely *C*_1_ = 0.164 m^−1^, *C*_2_ = 0.328 m^−1^, *C*_3_ = 0.492 m^−1^, and *C*_4_ = 0.656 m^−1^, were selected for the numerical analysis of the bending behavior of a standard cross-section with a curvature of *C*_0_ = 0.2 m^−1^. This analysis utilized linear–elastic ideal plastic and viscous plastic material models. The moment–curvature curves for the loading processes of these material models are illustrated in Figure 7. It is evident that the bending moment–curvature curves differ significantly between the two material models. This difference primarily arises from the fact that the bending moment during the loading process was mainly influenced by the applied stress. Specifically, the yield moment of the linear–elastic ideal plastic model for the section was lower. Consequently, under identical conditions of curvature and straightening, the linear–elastic ideal plastic model exhibited a lower bending moment compared with the viscoplastic material model. Therefore, the section governed by the linear–elastic ideal plastic model was more susceptible to elastic–plastic bending under such circumstances.

## 3. Mechanical Behavior of Hot Straightening of a Medium-Thick Plate Based on Viscoplastic Materials

**Figure 8:**
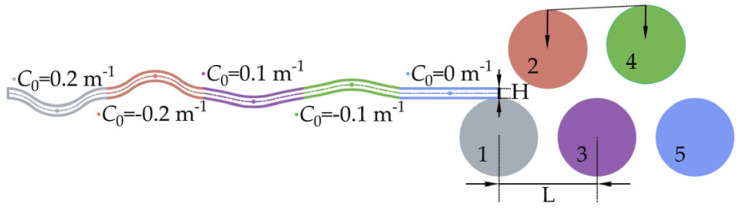
Schematic diagram of a 5-roll straightening process with multi-valued initial curvature.

In order to systematically analyze the mechanical behavior of the thermal straightening of medium-thick plates, an illustrative case of a five-roll thermal straightening process was examined. The analysis was based on a large deformation straightening technique and utilized numerical analysis corresponding to the material model discussed in the previous section. The large-deformation straightening process involved multiple instances of forceful bending (large deformation) to correct varying initial curvatures of plates, aiming to achieve a uniform curvature. This was followed by leveling, based on a standard method for straightening plates with a single curvature value. Empirical data from actual production was used to determine the rollback bending rates, as detailed in Table 4. These conditions included a horizontal roller distance (L) of 0.36 m, a thermal straightening temperature range (*T*_0_) of 450 °C to 650 °C, and a straightening speed range (*v*) from 0.4 m/s to 2 m/s. The plate was assumed to have a varied initial curvature (C_0_) falling within the range of [−0.2 m^−1^, 0.2 m^−1^], distributed randomly and continuously. The setup of the straightening machine and the initial curvature distribution are detailed in Figure 8, considering a plate section width (B) of 1 m and section height (H) of 0.02 m. Based on different thermal straightening conditions, the plate’s yield limit stress (*σ*_s_) determines the corresponding elastic limit moment *M*_t_ ($M_{t}=\frac{BH^{2}}{6}\sigma_{s}$) and elastic limit curvature *C*_t_ ($C_{t}=\frac{2\varepsilon_{t}}{H}$). To study the evolution of cross-section residual stress during large deformation processes, *σ*_cmax_ represents the maximum cross-section residual stress, while *σ*_cb_ denotes the value of the residual stress at the cross-section edges. Both of these quantities serve as parameters for evaluating the level of residual stress. Additionally, the maximum (C_C+_) and minimum (C_C−_) residual curvatures of the plate’s cross-section post-straightening define C_C+_ and C_C−_. The distribution interval of the residual curvature, denoted as {C_C_}, is equal to the absolute difference between C_C+_ and C_C−_. These factors are considered indicators of residual curvature level, with a smaller {C_C_} value indicating a higher degree of curvature uniformity across the plate.

### 3.1. Stress Evolution and Residual Stress Distribution in Typical Cross-Sections

**Figure 9:**
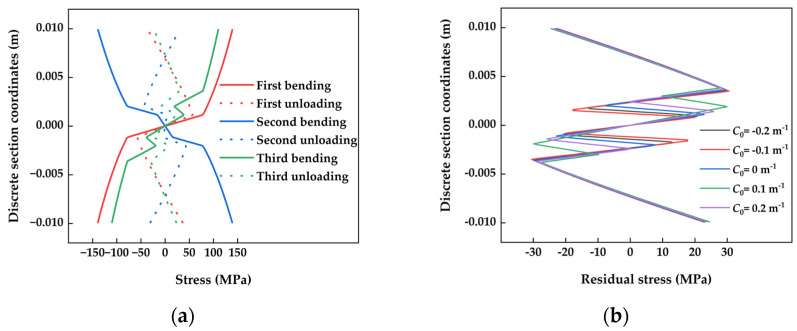
Stress evolution of hot-straightened cross-sections and post-straightened residual stress distribution under different curvatures. (**a**) Stress evolution in a typical cross-section during hot straightening; (**b**) comparison of post-straightening residual stress distribution in sections with varying initial curvatures.

The section with the largest initial curvature, denoted as *C*_0_ = −0.2 m^−1^, was chosen as a representative segment for the numerical analysis of stress evolution. The input parameters for the numerical calculations were set at *T*_0_ = 550 °C, *v* = 0.8 m/s, and the corresponding yield limit stress *σ*_s_ = 80.2 MPa. Based on the numerical analysis of the stress state across each straightening roller’s backbending, the evolution of stress in a typical cross-section is presented in Figure 9a. Comparing the three bending and unloading rebound processes, it was observed that the inelastic deformation region along the edge of the cross-section diminished due to the decreasing rate of backbending in the large-deformation straightening process. Consequently, the residual stress across the entire cross-section tended to homogenize, enhancing the distribution of residual stresses and aligning the residual stress levels between the edge and the middle. The distribution of residual stresses after straightening in sections of the plate with varying initial curvatures is illustrated in Figure 9b. The figure shows that the difference in residual stress distribution among sections with different initial curvatures was mainly concentrated in the middle. Sections with larger initial curvatures displayed higher residual stress levels in the middle region.

### 3.2. The Influence of Different Straightening Process Parameters on the Hot Straightening Characteristics of Medium-Thick Plates

The straightening temperature and speed are crucial process parameters in hot straightening, impacting the quality and efficiency of the post-straightening process. Therefore, investigating their effects on the hot straightening characteristics of medium-thick plates holds significant importance. Specifically, the experimental conditions for studying the influence of straightening speed on thermal straightening characteristics were chosen as follows: *T*_0_ = 550 °C, *v* = 0.4, 0.8, and 2 m/s. Likewise, the investigation focused on studying the impact of temperature on the thermal straightening characteristics under the following conditions: *v* = 0.8 m/s, *T*_0_ = 450, 550, 650 °C.

#### 3.2.1. Residual Stress Distribution in Typical Sections with Different Straightening Process Parameters

**Figure 10:**
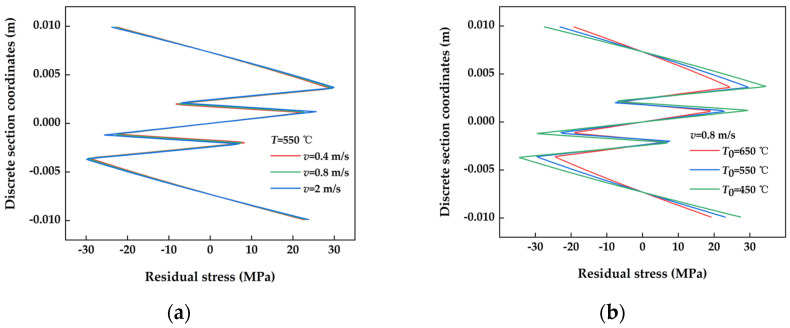
Post-straightening residual stress distribution in sections with various straightening process parameters. (**a**) Different straightening speeds; (**b**) different straightening temperatures.

The post-straightening residual stress distribution of the cross-section under different straightening speeds is displayed in Figure 10a. The distribution of residual stresses after straightening at various speeds appears to have been consistent and uniform. Overall, the residual stress profile and levels across the cross-section remained largely unchanged regardless of the straightening speed applied. This suggests that variations in straightening speed had minimal impact on the post-straightening residual stress within the cross-section. The residual stress distribution of the cross-section after straightening at various temperatures is illustrated in Figure 10b. The residual stress distribution and levels in the cross-section following the extensive straightening process were also found to be similar across different temperatures. However, variations in residual stress distribution among temperature conditions were primarily concentrated in the middle section. Notably, the middle part of the cross-section exhibited higher residual stress levels at lower temperatures, while the sides showed a corresponding decrease. This pattern indicates that the residual stress levels followed a consistent trend as temperatures decreased. Notably, the influence of temperature on residual stress distribution appears to have been more significant than that of straightening speed.

#### 3.2.2. Analysis of the Evolution of Residual Stress Parameters in Plates with Different Straightening Process Parameters

**Figure 11:**
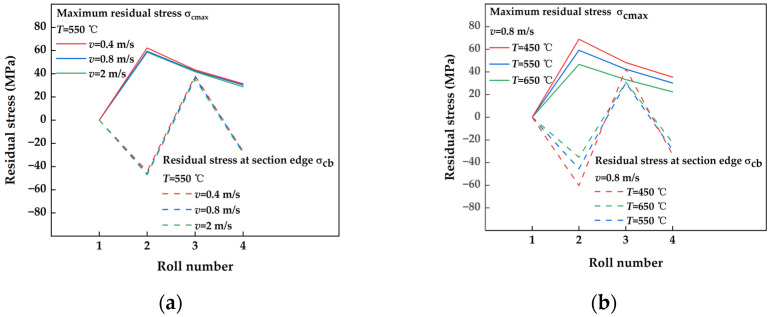
Variation of post-straightening residual stress coefficients in sections with different straightening process parameters. (**a**) Different straightening speeds; (**b**) different straightening temperatures.

The variations in cross-section residual stress parameters under different straightening speeds in the process of thermal straightening are illustrated in Figure 11a. The evolution of residual stress parameters in the figure revealed several key findings: the maximum residual stress *σ*_cmax_ of the cross-section decreased to a certain extent with the level of counterbending, aligning with the principle of decreasing counterbending rate in the large-deformation straightening process; the maximum residual stress *σ*_cb_ at the edge of the cross-section exhibited significant variability throughout the straightening process, maintaining a consistent pattern of change and evolution; the behavior of the cross-section under different straightening speeds remained consistently uniform throughout the straightening process. Notably, the maximum residual stress *σ*_cb_ at the section edge displayed a wide range of fluctuations during straightening, with a highly consistent pattern of evolution. The consistent characteristics of the residual stress parameter across various straightening speeds suggest minimal influence on the overall residual stress level within the cross-section. The changes in residual stress parameters under different straightening temperatures are depicted in Figure 11b, reflecting a similar evolutionary trend to that observed in Figure 11a, with higher straightening temperatures corresponding to lower values of cross-section residual stress parameters.

#### 3.2.3. Comparison of Changes in Residual Curvature of Cross-Sections with Different Straightening Process Parameters

**Figure 12:**
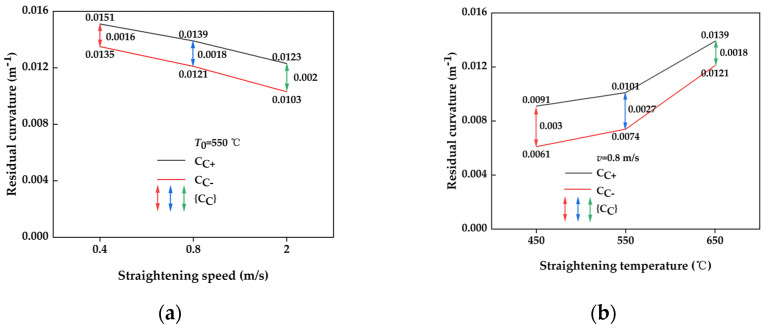
Comparison of residual curvature coefficients after cross-section straightening for different straightening process parameters. (**a**) Different straightening speeds; (**b**) different straightening temperatures.

Comparison of the residual curvature parameters of the post-straightening section of the plate at various straightening speeds is depicted in Figure 12a. The graph illustrates a gradual decrease in the maximum/minimum residual curvature Cc+/Cc− with an increase in straightening speed. Simultaneously, the residual curvature interval widened, albeit with a minimal increase. This observation suggests that the optimal straightening speed for the large-deformation straightening process, influenced by thermal effects, is relatively low. Moreover, it demonstrates that within the range of straightening speeds, the process is effective in correcting geometric defects. The comparison of the residual curvature parameter of the plate cross-section after straightening at different temperatures is illustrated in Figure 12b. A notable trend indicated in the graph is that as the straightening temperature increased, both the maximum and minimum residual curvatures Cc+/Cc− increased, resulting in a wider residual curvature interval. This trend indicates that within the specified temperature range, higher temperatures enhanced the efficacy of thermal straightening post-straightening.

## 4. Conclusions

In this paper, we present a cross-sectional continuous reverse bending model that considers temperature and straightening rate by analyzing the stress and curvature changes during the hot straightening process of medium-thick plates. To conduct our analysis, we utilized numerical calculation methods. Additionally, we constructed a viscoplastic material model that incorporated temperature and strain rate through fitting experimental data and utilizing mathematical methods. The study findings demonstrate the following:

(1) A mechanical model for continuously reverse bending straightening of plates was established using the difference method. Then, a bending solution model that considered the temperature gradient and strain rate gradient of the bending section was obtained using numerical analysis.
(2) Based on the experimental data from hot simulation, a fitting process was performed to establish the ontological relationship between the Q345 plate and the parametric equation of the material’s high-temperature elastic modulus. This model enabled a more accurate description of the stress–strain relationship during hot deformation.
(3) By analyzing and comparing the mechanical behaviors and bending characteristics of different material models, it can be seen that the loading stress distribution of the viscoplastic material model during the bending process was significantly different from that of the linear–elastic ideal plastic material model in the plastic section. However, the corresponding residual stress distribution was not significantly different, due to the influence of the material properties during rebound. The bending moment level of the linear–elastic ideal plastic model was significantly smaller than that of the former model, indicating that the former model was more prone to elastic–plastic bending. The bending moment level of the linear–elastic ideal plastic material model was obviously smaller than that of the viscoplastic material model, indicating that the corresponding section of the former was more susceptible to elastic–plastic bending.
(4) Based on a cross-sectional discrete difference model that considered temperature and strain rate, analysis was conducted on the straightening process of medium-thick plates under conditions of large deformation. The findings indicate that the temperature during straightening had a more pronounced impact on the overall process than the strain rate. Additionally, it was identified that a straightening scheme based on the decreasing reverse bending principle provided better control over the level of residual stress.
(5) Upon analyzing the large-deformation process scheme with varying straightening temperatures and strain rates, it was observed that higher temperatures resulted in smaller and more concentrated residual stress, which facilitated the straightening process. Conversely, higher strain rates resulted in more dispersed residual stress, making straightening more challenging. The large-deformation straightening process aligns with the capability to correct geometric defects within the suitable range of straightening temperatures and strain rates.

## Figures and Tables

**Table 1:** Chemical composition of Q345 steel (wt/%).

C	Si	Mn	P	S	Al	Cu	Ni	Nb
0.16	0.3	1.15	0.0172	0.084	0.0313	0.0232	0.0115	0.00268

**Table 2:** High-temperature simulation tensile test program.

Serial Number	Deformation Temperature T (°C)	$\mathrm{Strain}\;\mathrm{Rate}\;\overset{\cdot }{\varepsilon }$ (s^−1^)
1	450	0.01
2	450	0.1
3	550	0.01
4	550	0.1
5	650	0.01
6	650	0.1

**Table 3:** Initial stress values for various conditions.

Deformation Temperature T (°C)	$\mathrm{Strain}\;\mathrm{Rate}\;\overset{\cdot }{\varepsilon }$ (s^−1^)	*σ*_0.2_ (MPa)
450	0.01	140.546
450	0.1	155.460
550	0.01	80.683
550	0.1	89.245
650	0.01	46.318
650	0.1	51.233

**Table 4:** Plate large-deformation straightening theory of each roll’s backbending process parameters.

Roll Number	1	2	3	4
Curvature C_w_ (m^−1^)	0	0.5	−0.2	0.1

## Data Availability

Data are contained within the article.

## Nomenclature

*A*, *Q*	temperature regression coefficient
*a*, *b*, *c*, *d*	modulus of elasticity coefficient to be determined
*B*	a plate section width, m
*C*	bending curvature, m^−1^
$C_{C_{n}}$	the residual curvature of the section after the nth bending spring-back, m^−1^
*C*_C+_	the maximum residual curvatures of the plate’s cross-section post-straightening, m^−1^
*C*_C−_	the minimum residual curvatures of the plate’s cross-section post-straightening, m^−1^
*C_n_*	the curvature of the nth reverse bending, m^−1^
*C_t_*	the elastic limit curvature, m^−1^
*C_w_*	the loading curvature, m^−1^
*C*_0_	the initial curvature, m^−1^
*D*	the regression coefficient that depends on the grade of steel
*E*	elastic modulus, MPa
*H*	thickness of the plate, m
*I*	the section’s moment of inertia, kg·m^2^
*i*	the strain rate hardening index
*k*	temperature coefficient
*L*	the distance between the straightening rollers, m
$M^{\left(n\right)}$	the nth bending moment of the section, N·m
*M*_t_	the elastic limit moment, N·m
*m*	the strain hardening index
R^2^	the regression exponent
*T*(*z*)	the deformation temperature, °C
*T*_0_	the temperature of the bending neutral layer within the cross-section, °C
*t*	the time from the point of zero bending moment to the point of maximum bending moment, s
*v*	straightening speed, m/s
*z*	the coordinate of the cross-section along the thickness direction, mm
*ε*	strain
*ε_s_*	the ultimate yield strain of the section
ε*_t_*	the elastic limit strain
${\varepsilon_{c}}^{\left(n-1\right)}(z)$	the cross-section of the residual strain after the n −1th bending
$\overset{\cdot }{\varepsilon }$	strain rate, s^−1^
*σ*_cb_	the value of the residual stress at the cross-section edges, MPa
*σ*_cmax_	the maximum cross-section residual stress, MPa
${\sigma_{c}}^{\left(n-1\right)}(z)$	the cross-section of the residual stress after the n −1th bending, MPa
${\sigma_{e}}^{\left(n\right)}(z)$	the loading stress in the elastic deformation zone, MPa
${\sigma_{ep}}^{\left(n\right)}(z)$	the loading stress in the viscoplastic deformation zone, MPa
*σ_s_*	the ultimate yield stress of the section, MPa
${\sigma_{w}}^{n}(z)$	the bending stress produced during the nth bending process. MPa
${\sigma^{\left(n\right)}}^{\prime }(z)$	the rebound stress of the nth bending of the section, MPa
{C_C_}	the distribution interval of the residual curvature, m^−1^
